# Carding Behavior and Bearing Capacity of a Newly Developed Cylinder Card-Clothing Compatible with Cotton and Terylene Fibers by Nb Alloying of AISI 1090 Steel

**DOI:** 10.3390/ma17071511

**Published:** 2024-03-27

**Authors:** Weihua Gu, Fuguo Li, Youchang Cao, Qinchao Gao, Chengzhi Zhuo

**Affiliations:** 1 Geron Card Clothing Co., Ltd., Nantong 226009, China; guwh@geron-card.com (W.G.); gaoqc@geron-card.com (Q.G.); zhuocz@geron-card.com (C.Z.); 2 School of Materials Science and Engineering, Northwestern Polytechnical University, Xi’an 710072, China; 3 Joint R&D Center for Metallic Materials, Metallic Wire and Metallic Card Clothing, Xi’an 710002, China; 4 Texhong International Group Limited, Hong Kong, China

**Keywords:** carding, metallic card clothing (MCC), computational fluid dynamics (CFD), Nb alloying of AISI 1090 steel, wear resistance

## Abstract

Changing the metallic card clothing on a carding machine is costly when the spinning mills want to card different fibers from cotton to terylene or vice versa. This article proposes a newly developed cylinder card clothing compatible with cotton and terylene fibers by Nb alloying of AISI 1090 steel so that the spinning mills can change the type of fiber without changing the card clothing. Based on an idea developed from classical carding balance theory to study the adaptability of the cylinder card clothing for cotton and terylene fibers, the wall shear stress was used as the basis for compatibility analysis of carding behavior and bearing capacity with cotton and terylene fibers and as the focus of this study. Nb alloying of AISI 1090 steel showed good wear resistance in carding areas after heat treatment with high hardness above 840 Hv_0.2_ and extremely fine grain grade of 13.5 class, which increased about 25% compared to conventional 80 WV. The testing results in the spinning mills, including one cotton and two terylene fibers, showed good performance with this newly developed card clothing. In conclusion, the card clothing made of Nb alloying of AISI 1090 steel can handle different fibers with acceptable carding performance.

## 1. Introduction

Carding is a critical process in the production of yarn. During the process, fiber turfs are separated into paralleled single fibers to remove dust, impurities, and short fibers. The carding performance is strongly influenced by the application of various types of card clothing [1], especially metallic card clothing installed on the main cylinder and flat-top card clothing installed oppositely in a carding machine. In classical operating experiences, the card clothing types for cotton fibers differ from those for man-made fibers. This phenomenon is a drawback for spinning mills when they want to change the types of yarn repeatedly to quickly adapt to the market demands because of the high cost of changing card clothing due to extended machine downtime and expensive card clothing. The MAGNOTOP system [2] for the quick change of a flat top developed by Trutzschler can reduce the downtime to several hours to avoid the drawbacks and improve adaptability to fibers. However, the system was not cheap enough and could not be applied to most types of carding machines due to technical problems.

A cheaper method was developing a type of card clothing that could adapt to several different fibers without changing the card clothing. Before designing the unique card clothing, we should know how the card clothing works in the carding process.

A classical theory points out that the carding process was just the fiber transfer competition balance between the flat-top needles and the tooth tips of the cylinder card clothing. In their classical article [3,4,5], Singh and Swani proposed a value of pf to describe the ratio of the fiber load held between the flat-top needles and cylinder teeth in the carding state to the fiber load on the cylinder. The pf was defined as the ratio of the fiber load on the flattop and the fiber load on the surface of the cylinder.

In their opinion, better carding performance needs a higher pf. The experience told us that pf had limitations because too high of a pf could result in the fill-up of the flat top and shut down the machine. Once a type of card clothing was installed, the value of pf would be limited to a narrow range. The value may be suitable for cotton, but for man-made fibers, it could be too small to have good performance or too big to avoid the shut-down of the carding machine. This was why the spinning mill had to change the card clothing to adapt to different fibers. 

However, which dimension of the card clothing affected the pf was unclear. A Japanese engineer suggested that the working angle of metallic card clothing was the critical parameter that influenced the balance in the carding process [6]. So, card clothing with three different working surfaces on one tooth tip was invented. Thus, the range of pf was expanded. The top on the teeth gave a suitable pf range for cotton, and the middle gave another range for man-made fibers. In brief, the card clothing provided at least two different carding positions, one for cotton and one for man-made fiber. However, the truth was that only the fibers held at the top of the teeth would be well carded. The middle of the card clothing was not a good position for any fibers. Not only does the working angle impact the balance but also the carding position from the tips. The main reason for this pity was a lack of a systematic estimation method to describe the card clothing quantitatively.

Fortunately, with the development of simulation methods, the complex airflow between the narrow space of cylinder card clothing and flat-top needles can be well investigated. This will significantly help us understand the balance in the carding process, which could make the adaptable cylinder card clothing compatible with cotton and man-made fibers. 

Since the first attempt from RWTH Aachen University by Mahlmann I [7], computational fluid dynamics have been recommended as one of the essential methods to investigate carding progress. One of the reasons was that the airflow was so strong that it would influence the movement of the fibers, while the spacing between the cylinder and the flat top was too small to easily put any device for measurement [8]. The airflow drag that could be strong enough to pull a single fiber out of the turfs was then verified according to the simulation by Shanshan He in 2019 [9]. However, the simulation was needed to improve accuracy further to investigate more carding details because of its abstract and simple flat-top needles and rough mesh grid. The carding area was a complex geometry with too many faces, so the simulation consumption was always a big problem. Thus, our group applied a simplified carding area with a uniform flat-top needle model for simulation in developing double-tooth carding clothing in 2021 [10]. In that article, the airflow distribution around tooth tips was taken as a physical indicator to describe the carding performance of metallic card clothing. This indicated the development direction of high-output card clothing and was corroborated by several patents from other card clothing providers [11,12,13]. However, the geometry was still not accurate enough to distinguish between any match of the metallic cylinder card clothing and the flat-top needles. 

Due to the primary function of the card clothing being influenced by the shape, the wear resistance of the card clothing was significant. So, the material of the card clothing was an essential issue in designing the new metallic card clothing. Studies investigating the effect of alloying elements on the local microstructure and mechanical behavior have been carried out recently [14,15]. The development of steel in card clothing was slow because of the extreme property requirements of high ductility in its annealed state for drawing to wire less than Φ1 mm and extremely high hardness of more than 800 Hv_0.2_ after quenching. Tungsten and vanadium alloyed steel, also known as 80 WV in the field of textile equipment, has been chosen as the primary material for card clothing since the 1990s [16], and it is still applied to most high-end card clothing. This was a big drawback because of the increased wear resistance requirement in modern high-speed and output carding machines. The unclear wear mechanism between fiber and steel mainly caused the difficulty with the development of steel. Fortunately, the attempts at niobium alloying of high carbon steel showed that niobium could generate much finer grain size and increase the eutectoid content in the martensite, which could increase the wear resistance of steel when the fiber was chosen to be the grinding material, similar to the polishing process [17,18,19]. In this article, a first attempt of this method on AISI 1090 steel was carried out with the production and wear test of metallic cylinder card clothing, which showed an evident increase in the wear resistance.

The friction and wear of metallic card clothing is a significant problem in the textile industry. By adopting appropriate design and material manufacturing processes, the wear resistance of metallic card clothing can be effectively improved. Especially when a new design of metallic card clothing is compatible with different fiber materials, the above problems become more urgent. It is necessary to analyze and understand the carding behavior and bearing capacity of a newly developed cylinder card clothing from the perspective of simulation calculation so as to provide basic design parameters for materials and material processes and lay the foundation for explaining the deep mechanism of friction and wear of cylinder card clothing in the next step.

This article employed a novel and precise geometry to analyze a cylinder card clothing compatible with both cotton and terylene fibers through computational fluid dynamics. This approach posed numerous challenges, including mesh generation and fine-tuning the algorithm to ensure convergence. Wall shear stress was chosen as a metric to depict the equilibrium of fiber movement between the cylinder card clothing and the flat-top needles. In the following parts of the article, we demonstrate that the method shows the potential to estimate the carding balance quantitatively, in other words, pf. The wall shear ratio between the flat-top needles and the cylinder teeth show the potential to be a positive correlation quantity of pf.

Consequently, an adaptable cylinder clothing was developed by Nb alloying of AISI 1090 steel, renowned for its exceptional strength and wearability. This innovative cylinder card clothing boasts at least two distinct tooth shapes within a repeat section along the tooth wire, enabling fibers to occupy two positions for optimal carding balance in cotton and synthetic fibers. In the present study, the tooth shape design parameters and wear resistance of the tooth were thoroughly analyzed using this carding simulation method. The cylinder wire made from AISI 1090 steel with Nb alloying and new tooth shapes underwent testing in the spinning mills of Texhong International Group Limited. The findings reveal that this metallic card clothing could be utilized for both cotton and terylene fibers, yielding satisfactory sliver quality through mere adjustments to the carding parameters. This eliminated the need to replace the card clothing or incur excessive downtime. 

## 2. Design and Experiment

### 2.1. Metallic Card Clothing Design

**Figure 1:**
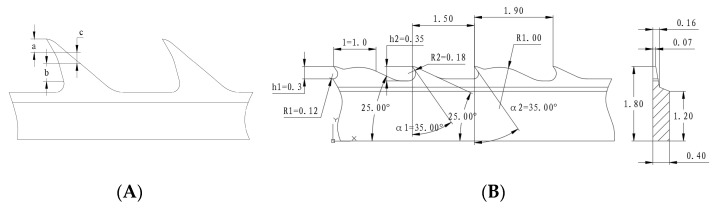
Tooth profiles of the card clothing: (**A**) The previous invention from the patent of JPH01306625A [14], (a) the carding position for cotton fibers, (b) the carding position for synthetic fibers, (c) the concave face between a and b; (**B**) the newly invented card clothing with two teeth, h1 and h2—the tooth depth, R1 and R2—the bottom arc, α1 and α2—the working angle, l—the humb length. A typical dimension is also marked in the graph.

Similar to the previous idea from Japanese engineers [16], the new card clothing was also invented with fewer than two positions for holding fibers on the working surface. Different from the previous invention, in the new card clothing, the two different positions would be located on two different teeth with different tooth profiles (Figure 1). The advantage of the new one is that fibers would always be held at the top area of the teeth so that the fibers have a higher probability of being caught up by a flat-top needle, which would help improve the carding performance. The theory here was verified with the invention of double-tooth card clothing, published in 2021 [10]. The newly designed cylinder card clothing seems to obey this rule worldwide from different card clothing providers, including famous European factories [11,12,13].

One of the difficulties with the design of the card clothing is how the card clothing can be made on classical rotary punching machines with few changes. This is important because rotary punching is the most efficient card clothing manufacturing process. Rotary punching is one of the punching technologies described in U.S. Pat. No. 6195843. The document is now included in Reference [20]. To meet the demand, the working angles of the different teeth are the same as each other (Figure 1). In other words, to ensure this card clothing could be manufactured in quantity, the angles α1 and α2 should preferably be the same. In conventional card clothing, the working angle of the cylinder card clothing for cotton is about 40°, while for terylene, it is about 20–25°. In this newly designed card clothing, the working angle was designed as 35° for compatibility. The risk of this angle design is that it would not have enough holding force to hold the cotton fiber or have too large a holding force that would lead to the terylene fiber being warped on the cylinder teeth. As an application-oriented design result, the holding force of the teeth can be distinguished not only by the working angle but also by the tooth depth and bottom arc. Tooth II (Figure 1) has a deep tooth depth and a small bottom arc for a higher holding force of the fibers, so it is the position for cotton fibers. The huge hump on the back of the teeth guides airflow to avoid the intertwinement from man-made fibers, which always has higher contact friction with metal surfaces. The end of the long man-made fibers are lifted by the airflow so that the flat-top needles can easily catch them. 

The PPSI (point per square inch) of the card clothing is another critical issue that needed to be carefully considered. Based on experience, the PPSI for cotton is always around 900–1000, while for terylene, it is about 700–860. The carding performance of the card clothing for cotton could be improved by shortening the tooth depth to make up for the loss caused by the reduction in PPSI. Finally, the tooth depth was designed to be 0.30–0.35 mm, and the PPSI was designed to be 850–950.

### 2.2. Material of Card Clothing

Since this new card clothing was designed to be compatible with different fibers, the robustness of the carding clothing needed to be improved to deal with a much more complex and severe environment. Not only would the shape of the teeth need to be controlled at a high level with few burrs even though the tooth shape is more complex than before, but also the wear resistance of the card clothing would need to be improved. This always means a definite improvement in the steel’s strength and toughness. According to the classical Hall–Petch relationship, a grain size refinement could be a suitable method. For the steel of card clothing, high carbon steel, for example, AISI 1070 and AISI 1080, have always been appropriate choices for producing medium-end card clothing with low cost and excellent ductility with a spheroidizing annealing state and high hardness after quenching. However, AISI 1090 would not be a good choice even though it has a potentially higher hardness because of its lousy ductility in the drawing of wire and teeth punching with a much higher risk of breakage. Hence, refining the grain size with the help of niobium with a micro-content of 0.03% and vanadium could be an excellent method to improve its ductility. The content of the newly developed steel is shown in Table 1 with the comparison of Nb alloying of AISI 1090 steel.

The steel was initially manufactured in a steel mill into Φ5.5 mm standard wire in a sorbite state, which was subsequently utilized for producing metallic card clothing. To remove the scale, the Φ5.5 mm wire underwent treatment with hydrochloric acid at a temperature of 60 °C. Following this, the steel wire was drawn down to Φ2.2 mm before being sent for normalizing. This normalization process involved heating the wire to approximately 780 °C using propane fire and subsequently cooling it with liquid lead. Later, the wire was air-cooled to room temperature. Several cold drawing steps further reduced the steel wire to Φ1.04 mm. Finally, the steel wire was introduced into a 4 m-high bell furnace for spheroidizing annealing and maintained at 700 °C for 8 h. The fine wire underwent a cold-rolling process to achieve the desired cross-section. Following rolling, the specially shaped wire was placed on a rotary punching machine to form the final tooth shapes. Subsequently, a high-frequency coil heated the card clothing to 750 °C in less than 1 s. Immediately after that, it was heated to 900 °C using a methane–oxygen flame, exceeding the austenite transformation temperature within 0.15 s. The subsequent quenching process was conducted online using an oil-quenching bath at room temperature, lasting approximately 4 s.

The teeth tips of the card clothing were then tested with a metallographic analyzer to identify the grain grade and with a microhardness tester to test the hardness. The teeth tips were tested to determine their wear resistance with a specially designed tester [21], which contained an abrasive disk made of soft cloth to simulate the fiber and a platform to install metallic card clothing, compared with other materials, including 80 WV for high-end card clothing.

### 2.3. Model Analysis by CFD

#### 2.3.1. Geometry Set-Up

**Figure 2:**
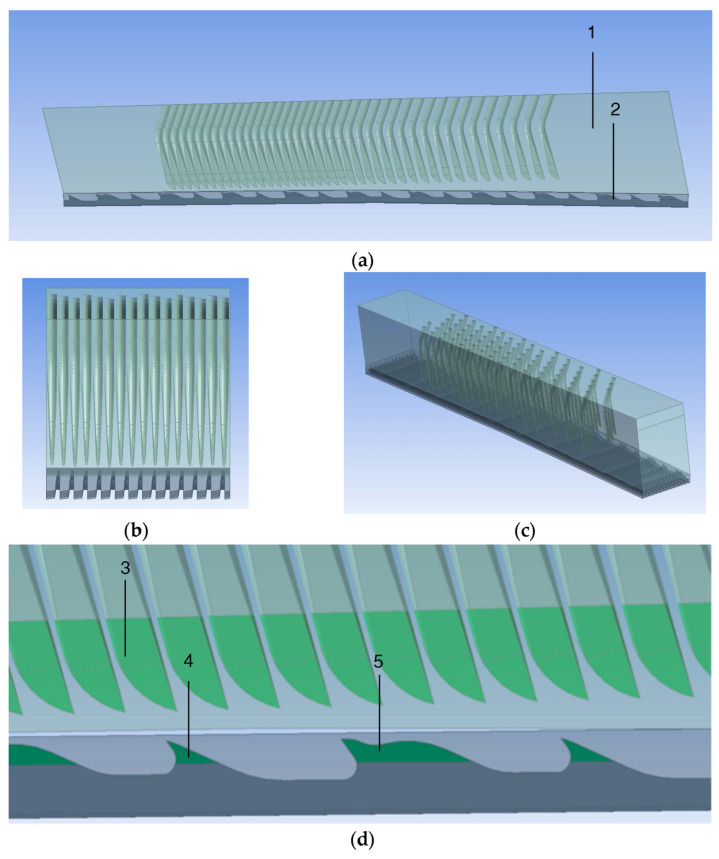
The 3D structure of the simulation airflow domain between flat-top needles and cylinder teeth: (**a**) from the front, 1—flat domain with actual non-uniform needle arrangement, 2—the cylinder wire domain; (**b**) from the right; (**c**) an overall view; (**d**) the split regions, 3—needle tips, 4—tooth I tips, 5—tooth II tips.

The geometric model of computational fluid dynamics is shown in Figure 2, with a simplified repeat unit between the cylinder and flat-top domains. The rotational/periodic symmetric boundaries can be regarded as an approximate model of the central carding region. Different from the previous study [10], the flat-top part could now represent a total repeat unit of any flat-top needle patterns, including uniform and non-uniform needle arrangements. The domain at two sides of the flat-top area restored the distance between two adjacent flat-top strips. The whole flat-top region was tilted slightly to consider the heel–toe difference, a classical carding parameter. The entire area took 1/120 of the circle with a degree of accuracy of 3.00°. Consequently, the cylinder region acquired a cambered shape featuring more than 200 teeth.

The flat-top needles and the cylinder teeth surfaces were then split into different parts to distinguish the functions of the regions. Figure 2 shows the areas involved that are in contact with the fibers in the carding process in green. There are three sections, named “needle tips”, “tooth I tips”, and “tooth II tips”. These surfaces have a much finer mesh because of their importance in carding, and these regions are also used to monitor the calculation steps.

**Figure 3:**
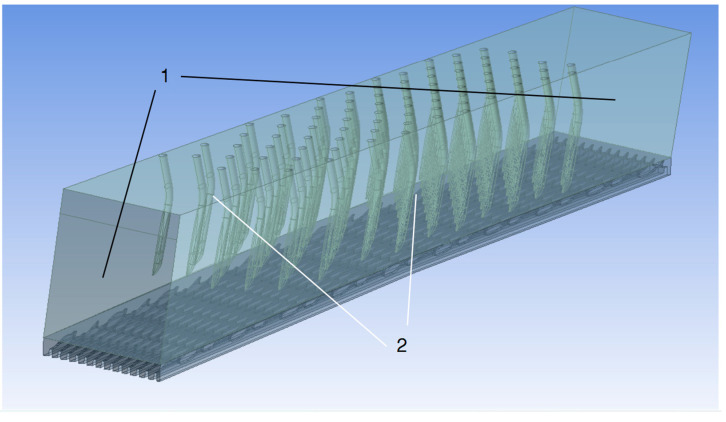
The boundary type of the simulation airflow domain: 1—the rotational/periodic symmetry plane group of the left and right surfaces; 2—the mirror symmetry plane group of the front and back covers.

The boundary types of the whole geometry of the simulation airflow domain are shown in Figure 3. The domain was closed without any airflow entrances or exits. The outer surfaces were all mirror and rotational/periodic symmetry planes. This was the nearest simulation condition compared to the actual carding situation. Ensuring all the corresponding symmetry planes had the same surface area was essential. Only in this way could the following calculation always converge with the residual error of less than 10^−4^ in fewer than 500 steps with steady-state simulation. 

#### 2.3.2. Mesh Generated and Independence Checking

The mesh was generated by Fluent meshing software (Version 2021R3) in the form of a polyhedral. Then, the mesh quality was improved by the “Improve Volume Mesh” tools with a target element quality of more than 0.2. The reason for using a polyhedral was the better convergence performance, lower calculation consumption, and faster generalization of high-quality mesh elements [22]. The classical tetrahedral mesh was tested at the beginning of the present research. However, it was abandoned after several tests because of its lousy convergence stability and too-high calculation consumption. The brick mesh could not be applied in this study because of the complexity of the fluid domain. 

**Figure 4:**
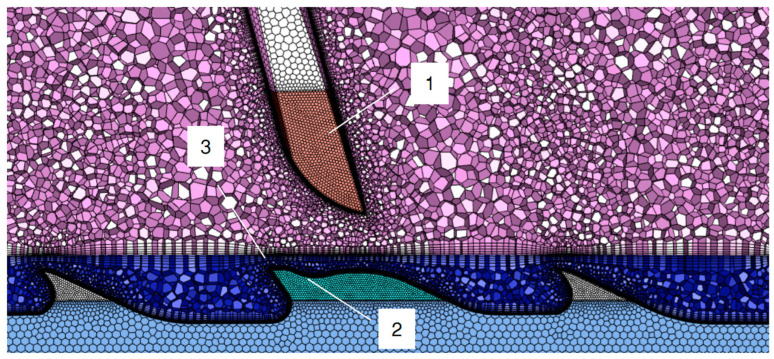
Typical mesh grid in polyhedral form with delicate boundary layers: 1—flat-top needle region with refined mesh and boundary layers; 2—cylinder teeth region with fine mesh and boundary layers; 3—refined mesh between the interface of the two fluid domains.

The cross-section of this type of generated mesh is shown in Figure 4. The interfaces of the flat top and the cylinder fluid domain were filled with fine mesh grids, as they aided in reducing the residual error of the carding simulation. This enhancement may also have contributed to the resolution of the intricacy and intensity. The surfaces of the flat-top needles and the metallic cylinder teeth were filled with ten boundary layers. The test shown in Table 2 yielded the grid independence-checking results of different boundary layers. Table 3 shows the grid independence checking of the needles’ and the teeth’s surface, including coarse and fine mesh settings. The difference in monitored results was less than 3.5% between the medium and fine mesh. Finally, the total mesh elements were about 15–22 million, which took over 12 h of calculation on a small server with 64 CPU cores. It should be noted that the geometry used in the grid independence tests was a draft design, which showed a minor difference from the final tooth shape calculated below.

#### 2.3.3. Adjustment of Algorithm and Convergence Test

**Figure 5:**
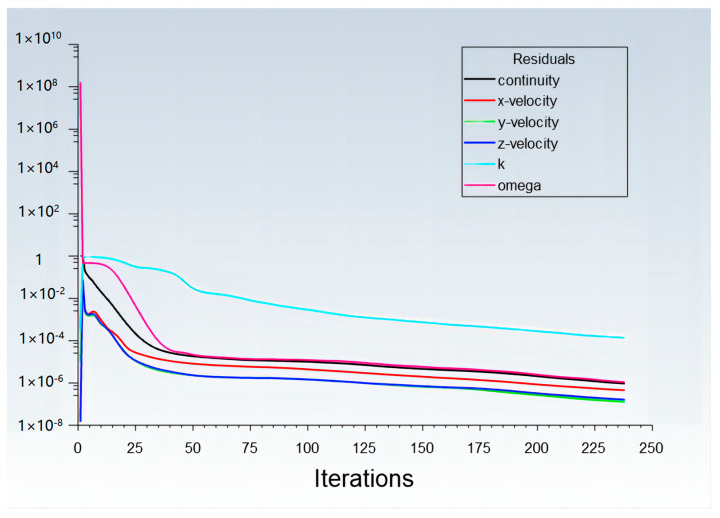
The typical convergence curves of a single test with low residual error.

The simulation was carried out by Fluent software (Version 2021R3) with its MRF (Multiple Reference Frame) models, and the simulation was set to be in a steady state. The transient simulation with SMM (Sliding Mesh Model) could also be carried out with the same geometry and meshes. But due to the fact that it was easy to converge with the steady-state results as its initial conditions, the transient simulation took too much time to finish a single calculation test for more than several days, and the transient simulation showed little difference in the physical data, which we were concerned about based on the steady-state simulation. As a result, the steady-state simulation with the MRF model became the first choice in this study. The turbulence model was chosen as the k-ω SST model, with room temperature air as the fluid material. Because of the significant number of mesh elements, the convergence of this model was a big problem at the beginning of the project. After several adjustments, the double precision option in Fluent needed to be opened even if it would cost massive memory usage. The algorithm options are shown in Table 4. As a result, the simulation could always be stably convergent with a residual error of less than 10^−4^ in fewer than 500 steps with a steady state. The convergence step depended on the structures of the card clothing, varying between 150 and 500. Figure 5 shows the typical convergence curves of a single test. 

### 2.4. Experiments in Spinning Mills

The tests of the new card clothing were carried out in one of the spinning mills at Texhong International Group Limited. The new card clothing was applied on three fibers, including cotton and terylene. The carding results were compared with the average quality of the spinning mill to estimate the carding performance of the new card clothing. The carding process parameters, including the carding gauge and cylinder speed, were adjusted to meet better carding performance, and the carded sliver was then tested.

## 3. Results Analysis and Discussion

### 3.1. Material and Wear Testing Results

**Figure 6:**
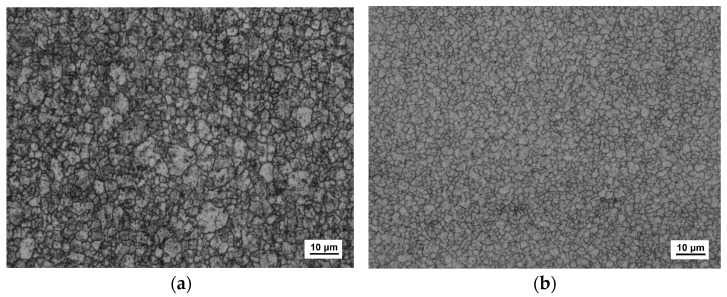
The grain structure diagrams of the steel on the tooth tips of the card clothing: (**a**) AISI 1090; (**b**) Nb alloying of AISI 1090.

**Figure 7:**
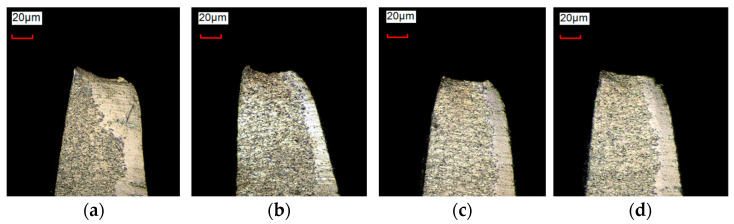
The burrs on the tooth tips of the card clothing: (**a**) AISI 1070; (**b**) AISI 1080; (**c**) 80 WV; (**d**) Nb alloying of AISI 1090.

Figure 6 shows the grain size before and after the addition of niobium. The grain grade was improved to about 13.5, which was fine enough to handle the deformation during the process. The cut edge of the card clothing was also identified with an optical microscope, and the results showed a smaller burr after punching compared with other conventional materials (Figure 7). 

**Figure 8:**
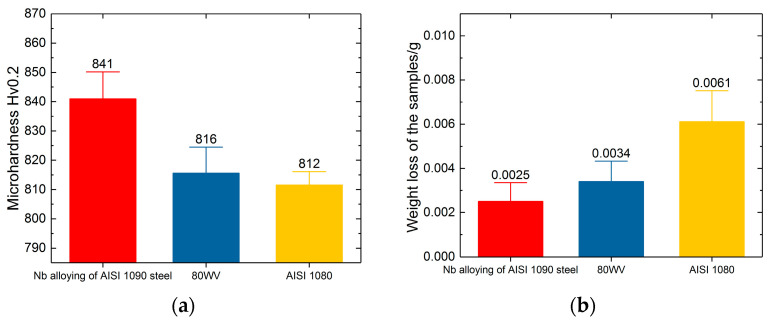
The wear resistance tests of the card clothing: (**a**) the hardness tests of the teeth tips; (**b**) the weight loss tests with a specially designed tester.

The hardness test results showed a noticeable improvement in the new steel, with at least 20 Hv_0.2_. As a result, the wear resistance of Nb alloying of AISI 1090 steel was also improved by about 25% compared with 80 WV, which showed less weight loss in the wear test lasting about 9 h, with five examples for each material (Figure 8). The 80 WV always had a grain grade of 13 with suitable heat treatment because of the micro alloyed vanadium and tungsten, so the wear resistance increased a lot compared with carbon steel without alloyed elements like AISI 1080. The Nb alloying of AISI 1090 steel had a slightly smaller grain size and a higher hardness, so the wear resistance was also improved. These results show that a suggestion to improve the wear resistance of steel for card clothing is that a higher hardness and a finer grain size could be a better choice for the mechanism of wearability with high strength and toughness. 

According to the Hall–Petch effect, the hardness and toughness of the steel would be improved with refinement of the grain. Thus, the wear resistance of the steel would be enhanced. However, the average grain size of the AISI 1090 was about 2–3 μm, approaching the boundary where the Hall–Petch effect could be practical and showing a better wear resistance than expected. This meant there could be an unknown wear mechanism for the AISI 1090 steel. The wear mechanism could have a relationship with the size of soft abrasive particles, for the diameter of the fiber was about 10 μm, much smaller than common abrasive particles. It was still a problem to explain why soft materials could wear down hard materials. Recent research [23] showed that the detailed arrangement of the metallographic structure and the contact modes could significantly impact the wear resistance. The addition of Nb to AISI 1090 may have a similar mechanism, which should be further studied. Anyhow, Nb alloying AISI 1090 may be a good choice when the fiber is an abrasive particle in the textile industry.

### 3.2. Simulation Results with Conventional Card Clothing

**Figure 9:**
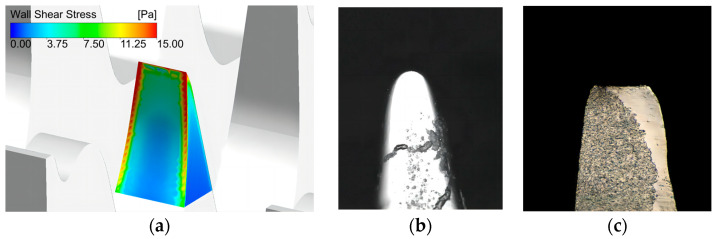
Wall shear stress distribution on the working surfaces of the card clothing and the wear distribution of it: (**a**) wall shear stress distribution; (**b**) worn tooth tip of cylinder card clothing; (**c**) new tooth tip.

This study selected wall shear stress as the physical quantity to describe the hold force of the carding elements, an indistinct experienced concept. The reason was that the wall shear stress had the same distribution as the wear on the surface of the containers in some references [24]. The wall shear stress distribution on card clothing seemed similar to the wear distribution (Figure 9), and the wall shear stress implied the carding position and changes in carding position in the simulation analysis. The higher the contact friction force between the fibers and tooth surface, the higher the loss of tooth tips because of the wear. This way, wall shear stress can be selected to act as a physical indicator to describe the holding force in the carding process.

Table 5 shows several matches of the card clothing applied in the spinning mills. The rules of the model name of each metallic card clothing can be found in the FZ/T 93038-2018 national standard of China [25]. MCH55/AC2040 × 01740 is a classical match that was used for decades due to its stable performance. However, this match always has good but not excellent performance with relatively higher neps in the sliver in recent years. MCBH58/AC1745 × 01835 was developed to obtain extremely low neps in the yarn. The wall shear stress ratio was raised between the flat-top and cylinder teeth, which showed that the fibers carried by the cylinder teeth were more likely to be caught by flat-top needles. The mechanism of the carding performance improvement was similar to that in the development of double teeth. The matches in the terylene were identical. Classical AC2520 × 01560 had lower breaking stress of the yarn with higher neps in the sliver, according to the carding result from a spinning mill in China. The carding parameters in Table 5 were all true and were carefully collected from real and typical spinning mills. 

This way, the development direction of the card clothing seemed to be clear. For the carding of cotton, the ratio of shear stress needed to be between 0.9875~1.2010, while for terylene, it needed to be between 0.8450~1.0570. A higher ratio limit in these ranges meant better performance. So, the balance of fiber transfer between the flat top and the cylinder can be described in mathematical values. The ratio of cotton and terylene was different, but they still had overlaps. The excellent performance in MCBH40S/AC1730 × 01550 and the good performance within an acceptable range in MCH 55/AC2040 × 01740 had a similar ratio of around 1. In a sense, this was the physical basis of the invention of the adaptable cylinder card clothing.

### 3.3. Simulation Result of the Adaptable Cylinder Card Clothing

The results show that the ratios of the wall shear stress between the flat-top needles and the cylinder teeth were not influenced by the cylinder speed. The competition between the flat-top needles and cylinder teeth was changed a little by adjusting the cylinder speed. This meant that the spinning mills could flexibly adjust the cylinder speed depending on their output and the yarn-quality requirement. The increased wall shear stress on the cylinder tooth tips and flat-top needle tips indicated a higher interaction with the fiber turfs, meaning a better opening effect, and could result in a better performance if the breakage of fibers was well controlled. However, as a disadvantage, the tooth and needle would suffer more wear and have a shorter service life at high cylinder speed. These inferences concluded by the simulation results were similar to the operation experiences concluded by engineers. The carding gauge did not affect the ratio of wall shear stress between flat-top needles and cylinder teeth. Consequently, these results imply that once the card clothing was determined, the fiber would be restricted to a limited number of similar types. Therefore, in conventional design, it is necessary to alter the card clothing if the fiber is changed from cotton to terylene.

In Table 6 and Table 7, the ratios were around 1 and 1.2, similar to the simulation results in Table 5, with cotton as the carding fiber. Tooth I in the card clothing yielded the standard carding performance, for its ratio was near 1, while tooth II performed better. For the carding of terylene, the results were different. Tooth I performed well, but tooth II needed to be taken care of. The ratio was a little bigger than the matches mentioned in Table 5. This meant the breaking of the balance. The cylinder teeth took too small a part in the carding, so the performance was not as good. There was better performance for tooth I and worse performance for tooth II, so the carding performance was still acceptable. As a result, this type of carding clothing could be acceptable in the cotton carding process, with good carding performance.

The design of card clothing is tailored to enhance cotton’s performance due to the varying components of its raw material. Cotton typically contains significantly more impurities compared to synthetic fibers, necessitating meticulous carding.

The results also showed the possibility of a single-tooth shape card clothing design with a ratio of nearly 1. Therefore, the performance of cotton would be common and terylene would be better. However, as mentioned above, the spinning mill is always more concerned about the quality of the cotton yarn, whether ring spinning or rotor spinning, because the quality of cotton yarn has an essential impact on the trading value. As a result, this design method is particularly suitable for specific target applications and unique environments and may not be widespread in the present climate. 

### 3.4. Results of the Wear Resistance

**Figure 11:**
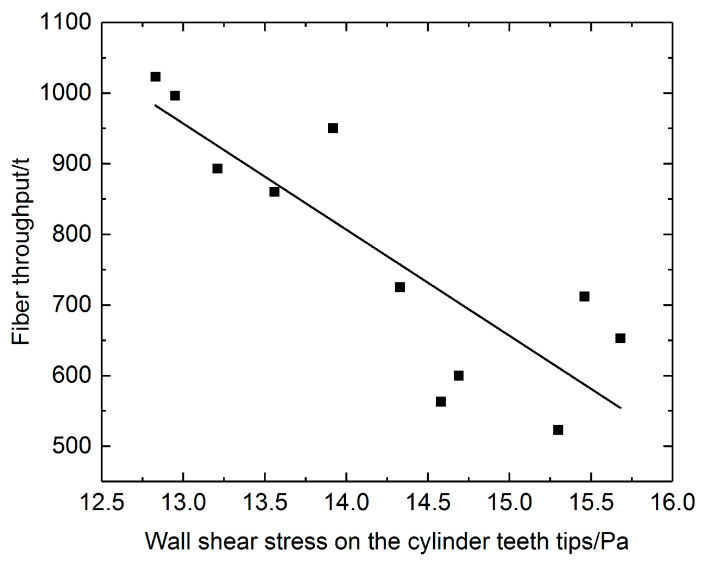
An approximate anticorrelation between average wall shear stress and the total fiber throughput during the whole service life. (■ A sample point of the service life provided by spinning mills; the line was an approximate fit).

Figure 11 shows an approximate anticorrelation between the average wall shear stress and the fiber throughput. After gathering the information from several spinning mills producing similar cotton yarn with similar raw material and the same yarn-forming process, the average wall shear stress calculated from the simulation showed an approximate anticorrelation between the life span of the card clothing measured by the fiber throughput. The higher the wall shear stress was, the lower the life span the card clothing could acquire. However, the quantity relationship was never built because too many factors could have an impact on the life span of card clothing. This anti-relationship is just a glance at the wear mechanism in the carding process. Still, the dominant role of soft materials in the shear stress of hard materials provides theoretical support for this. In the article, the wall shear stress was considered the holding force of the teeth tips, and it was also an essential factor that influenced the carding performance. In general, a higher carding force could yield better carding performance. It was interesting to find that the life span of the card clothing and the carding performance were contradictory, and this is a common phenomenon in most industrial processes.

### 3.5. Results in Spinning Mills

The test results of the yarn quality in spinning mills are shown in Table 8. In the first two types of fibers, including cotton 40S and terylene 16S, the adaptable cylinder card clothing showed better results than the average level in the spinning mill, regardless of CV%, neps, and A1 yarn defects. However, the neps increased when the fiber was changed to terylene 40S. This spinning mill was more concerned with A1 yarn defects, so the results were still acceptable. In conclusion, the better performance for cotton and the acceptable performance for terylene are consistent with the simulation results.

The reason for the excellent performance of the card clothing is that it yielded two ratios of wall shear stress, meaning two platforms for exchanging fibers between flat top needles and the cylinder teeth. Conventional card clothing has only one ratio, which is only suitable for several fibers. What is good for cotton is bad for terylene.

The parameters for any fiber were almost the same between the conventional and adaptable card clothing listed in Table 8. However, the carding process parameters were quite different between cotton and terylene, including the carding gauge and the speed of the cylinder, and they were all within the scope of simulation calculations and design considerations.

Another factor that needed to be considered was whether the types of flat tops were the same. If the types were the same, according to the beginning of this article, the problem of changing fibers without changing the card clothing could be solved. However, this test was imperfect because of a minor difference in the flat-top types for about 3% PPSI with the same needle arrangements. If the yarn quality was not so strict, the problem seemed to be solved by this newly designed cylinder card clothing.

However, the reason for the relative medium difference between terylene 16S and 40S was unclear. Perhaps the carding parameters should be further investigated with the help of the simulation method and more experiments in the spinning mills, and these will be carried out in subsequent work. The adaptable cylinder card clothing is now being utilized in spinning mills in Vietnam, with a scale of hundreds, for robustness testing.

### 3.6. Discussion about the Carding Simulation

The simulation of the carding process still has three critical problems worth considering. The first problem is the accuracy of the simulation. The airflow around the teeth and needles has a Reynolds number of 1000–3000, where the transition from laminar to turbulence may happen. The transition simulation is still a complex problem in CFD because of too many unexpected flow behaviors. The shape of the airflow domain infinitely influenced the transition model, so the applied standard k-ω SST model in this article had the risk of being inaccurate.

The second problem is the simulation of fibers for too much consumption in the calculation. The discrete element method could be used to describe the fiber behavior in the airflow coupled by CFD [26,27,28]. However, it was still too complex to simulate the carding process in the research of industrial application orientation at the enterprise level.

The third problem was how to analyze the results even better. How to create a correspondence between the calculated physical quantity and the carding performance is a crucial problem. In this article, the calculated results were associated with the carding performance by extending traditional carding theory [3] using the so-called carding balance as the bridge. As discussed in this article, the wall shear stress of needles and teeth offers a promising avenue for soft materials in the shear stress of worn hard materials. Additionally, the velocity distribution provides another example of the development of double teeth [10]. More and more related physical quantities would be found to describe the carding process if we continue to focus on improving the simulation model. Then, only by obtaining accurate simulation results can we provide a basis for explaining the deep mechanism of the friction and wear of cylinder card clothing made of Nb alloying of AISI 1090 steel in the next step.

## 4. Conclusions

In this article, a new card clothing compatible with cotton and terylene fibers by Nb alloying of AISI 1090 steel was successfully developed. Several conclusions were drawn, as follows:

1. The carding mechanism was explained quantitively by computational fluid dynamics. The wall shear stress ratio between the flat-top needles and the cylinder teeth was a key parameter to estimate the carding performance. The ratio was significantly affected by the shape of the card clothing other than the process parameters of carding, showing why the spinning mills must change the classical card clothing to adapt to different fibers.
2. The wear trend of the card clothing could be quantitively described by the simulation. The results showed good relevance to the Archard law. It was the first method to evaluate the wear of the card clothing during the design step for card clothing. With this method, the service life of card clothing under different situations could also be estimated.
3. An Nb alloying of AISI 1090 steel was developed to improve the wear resistance of the card clothing. Nb alloying of AISI 1090 steel showed good wear resistance in carding areas after heat treatment, with a high hardness above 840 Hv0.2 and an extremely fine grain grade of 13.5, which increased about 25% compared to conventional 80 WV. The wear resistance of the card clothing could be enhanced by the refinement of the grain size and the improvement of the hardness. This result would be an example of the research on soft abrasive wear.

## Figures and Tables

**Figure 10:**
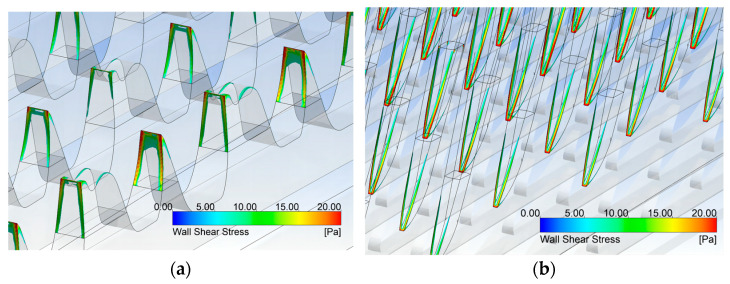
The sampling method of the wall shear stress: (**a**) tooth tips; (**b**) needle tips.

**Table 1:** The main chemical composition of different steels.

Elements Weight Content/%	Fe	C	Si	Mn	P	S	Cr	Ni	Cu	V	Nb
New	97.54	0.90	0.22	0.68	0.013	0.012	0.09	0.21	0.17	0.14	0.028
AISI 1090	97.65	0.91	0.23	0.67	0.015	0.011	0.11	0.22	0.16	0.02	0.0

**Table 2:** Grid independence checking of the number of boundary layers *.

Test No.		1	2	3
Number of boundary layers		5	8	10
Total mesh elements (million)		12.07	15.52	17.84
Monitored wall shear stress (Pa)	Needle tips	12.72	12.60	12.67
	Tooth I tips	11.40	11.40	11.45
	Tooth II tips	9.81	9.80	9.96

* The calculation carding parameters: cylinder speed 420 rpm (revolutions per minute); carding gauge 0.21 mm; heel–toe difference 0.56 mm; sampling area 0.0159 mm^2^ for each tooth and 0.0641 mm^2^ for each needle. The area strongly affected the results, so the area must be stated clearly.

**Table 3:** Grid independence checking of the surface mesh density *.

Test		Coarse	Medium	Fine
Surface mesh Size (mm)	Tooth tips	0.0211	0.0150	0.0106
	Needle tips	0.0282	0.0200	0.0141
Total mesh elements (million)		11.63	17.84	29.52
Monitored wall shear stress (Pa)	Needle tips	12.20	12.67	13.08
	Tooth I tips	11.04	11.45	11.75
	Tooth II tips	9.24	9.96	10.25

* The calculation carding parameters: cylinder speed 420 rpm; carding gauge 0.21 mm; carding heel–toe difference 0.56 mm; sampling area 0.0159 mm^2^ for each tooth and 0.0641 mm^2^ for each needle. The area strongly affected the results, so the area must be stated clearly.

**Table 4:** The algorithm options for stable convergence.

**Spatial Discretization**	
Pressure	standard
Momentum	first-order upwind
Turbulent kinetic energy	first-order upwind
Specific dissipation rate	first-order upwind
**Relaxation Factors**	
Turbulent kinetic energy	0.4
Specific dissipation rate	0.4

**Table 5:** Matches of the card clothing with their simulation results.

Fiber Type	Carding Process Parameters	Type of Card Clothing		Average Wall Shear Stress of Tips and Ratio/Pa *		
		Flat Top	Cylinder	Flat Top	Cylinder	Flat Top/Cylinder
Cotton	Cylinder speed: 400 rpm Carding gauge: 0.21 mm	MCH55	AC2040 × 01740	12.67	12.83	0.9875
		MCBH58	AC1745 × 01835	11.02	9.175	1.2010
Terylene	Cylinder speed: 420 rpm Carding gauge: 0.21 mm	MCBH40S	AC1730 × 01550	13.70	12.96	1.0570
		MCBH45	AC2520 × 01560	15.32	18.13	0.8450

* The sampling area was 0.0159 mm^2^ for each tooth and 0.0641 mm^2^ for each needle (Figure 10). The area strongly affected the results, so the area must be stated clearly.

**Table 6:** The simulation results of the new adaptable cylinder card clothing with different cylinder speeds and the same carding gauge.

Cylinder Speed (rpm)	Type of Card Clothing		Average Wall Shear Stress of Tips and Ratio/Pa *				
	Flat Top	Cylinder	Flat Top	Cylinder Tooth I	Flat Top/Cylinder Tooth I	Cylinder Tooth II	Flat Top/Cylinder Tooth II
420	MCH55	New developed AC1835 × 1740 (tooth depth: 0.35 and 0.30 mm; PPSI: 950)	12.41	11.49	1.08	9.96	1.25
440			13.36	12.34	1.08	10.77	1.24
460			14.65	13.27	1.10	11.61	1.26
480			15.69	14.19	1.10	12.49	1.26
500			16.73	15.14	1.10	13.37	1.25

* The sampling area was 0.0159 mm^2^ for each tooth and 0.0641 mm^2^ for each needle. The area strongly affected the results, so the area must be stated clearly. The carding gauge of this simulation was 0.21 mm.

**Table 7:** The simulation results of adaptable cylinder card clothing with different carding gauges and the same cylinder speed.

Carding Gauge (mm)	Type of Card Clothing		Average Wall Shear Stress of Tips and Ratio/Pa *				
	Flat Top	Cylinder	Flat Top	Cylinder Tooth I	Flat Top/Cylinder Tooth I	Cylinder Tooth II	Flat Top/Cylinder Tooth II
0.30	MCH55	New developed AC1835 × 1740 (tooth depth: 0.35 and 0.30 mm; PPSI: 950)	11.84	11.03	1.07	9.68	1.22
0.28			12.04	11.21	1.07	9.84	1.22
0.25			12.16	11.30	1.08	9.83	1.24
0.23			12.33	11.42	1.08	10.04	1.22
0.21			12.41	11.49	1.08	9.96	1.25

* The sampling area was 0.0159 mm^2^ for each tooth and 0.0641 mm^2^ for each needle. The area strongly affected the results, so the area must be stated clearly. The cylinder speed was 420 rpm.

**Table 8:** Yarn quality with different types of fibers.

Type of Fibers	Cylinder Card Clothing	CV%	Neps (+140%)	Neps (+200%)	A1 Yarn Defects
Cotton 40S (machine-picked, Xinjiang, fiber length <32 mm)	Conventional	8.24	12.5	5.5	83.1
	Adaptable	8.28	8.5	1.5	39.2
Terylene 16S (Sateri^®^, 1.33dtex, 38 mm, white)	Conventional	8.08	17	2	126
	Adaptable	7.72	9	0	36.2
Terylene 40S (Sateri^®^, 1.33dtex, 38 mm, white)	Conventional	11.51	122	14	354.2
	Adaptable	11.65	163	20	273.9

## Data Availability

Data are contained within the article.
